# Effects of Adding Corn Dried Distiller Grains with Solubles (DDGS) to the Dairy Cow Diet and Effects of Bedding in Dairy Cow Slurry on Fugitive Methane Emissions

**DOI:** 10.3390/ani4040767

**Published:** 2014-12-09

**Authors:** Daniel I. Massé, Guillaume Jarret, Chaouki Benchaar, Fadi Hassanat

**Affiliations:** Dairy and Swine Research and Development Centre, Agriculture and Agri-Food Canada, 2000 College Street, Sherbrooke, QC J1M 0C8, Canada; E-Mails: jarretguillaume@yahoo.fr (G.J.); chaouki.benchaar@agr.gc.ca (C.B.); fadi.hassanat@agr.gc.ca (F.H.)

**Keywords:** beddings, corn DDGS, dairy, manure, methane, storage

## Abstract

**Simple Summary:**

The objectives of this experiment were to investigate the effects of adding corn DDGS to the dairy cow diet as well as the bedding types (wood shavings, straw or peat moss) on manure fugitive CH_4_ emissions. The incorporation of DDGS in the diet has increased manure methane emission by 15% and the use of peat moss as bedding has increased manure methane emission by 27%.

**Abstract:**

The specific objectives of this experiment were to investigate the effects of adding 10% or 30% corn dried distillers grains with solubles (DDGS) to the dairy cow diet and the effects of bedding type (wood shavings, straw or peat moss) in dairy slurry on fugitive CH_4_ emissions. The addition of DDGS10 to the dairy cow diet significantly increased (29%) the daily amount of fat excreted in slurry compared to the control diet. The inclusion of DDGS30 in the diet increased the daily amounts of excreted DM, volatile solids (VS), fat, neutral detergent fiber (NDF), acid detergent fiber (ADF) and hemicellulose by 18%, 18%, 70%, 30%, 15% and 53%, respectively, compared to the control diet. During the storage experiment, daily fugitive CH_4_ emissions showed a significant increase of 15% (*p* < 0.05) for the slurry resulting from the corn DDGS30 diet. The addition of wood shavings and straw did not have a significant effect on daily fugitive CH_4_ emissions relative to the control diet, whereas the addition of peat moss caused a significant increase of 27% (*p* < 0.05) in fugitive CH_4_ emissions.

## 1. Introduction

Manure generated by livestock operations contributes to greenhouse gas (GHG) emissions. Methane (CH_4_), one of the principal agricultural greenhouse gases, is produced by enteric fermentation in ruminant animals [1,2] and by fugitive methane emission that occurs during anaerobic degradation of manure in livestock buildings and manure storage facilities [3,4,5,6,7]. The level of uncontrolled fugitive CH_4_ emission during manure storage is affected by environmental factors such as storage temperature [8,9], storage duration [10], manure composition and bedding content [10,11,12]. Environmental legislation and public concern about the environmental footprint of livestock productions have increased pressure on producers to take measures to reduce atmospheric and environmental pollution. Among the measures proposed to reduce environmental pollution from the livestock sector, animal nutrition has the strong potential to reduce enteric CH_4_ emissions, and anaerobic digestion allow the capture of fugitive GHG emissions and production and valorization of green energy. The addition of fat to a diet reduces or eliminates protozoa as well as methanogen bacteria in the rumen, resulting in decreased CH_4_ emissions and a shift in hydrogen sink through bio-hydrogenation via propionate production [13,14]. The recent increase in the availability of biofuel by-products, such as corn dried distillers grains with solubles (DDGS) which are rich in fat, proteins and fibers is increasing the interest in the use of these by-products in animal production. It has been used in beef diets with a view to reducing enteric CH_4_ emissions [15]. The extent of the degradability of DDGS fats, proteins and fibers in the animal rumen and gut will have an impact on the excreta composition and might result in a decrease or increase of fugitive methane emission levels from manure storage structures [16]. This experiment used an integrated approach to assess enteric CH_4_ and manure CH_4_ emissions related to dairy diets. It has been reported [17] that adding 20% wheat DDGS to pig diets modified manure quantity and characteristics and increased fugitive CH_4_ emissions during slurry storage. Moreover, since bedding is used in most dairy barns, there is a need for scientifically sound data on fugitive CH_4_ emissions from raw manure produced by cattle fed different diets as well as on the mixture of raw manure and beddings. Bedding usually consists of straw or wood shavings. Due to the low availability of wood shavings and the high cost of straw, some producers are showing an interest in peat moss. Within this context, the objectives of this experiment were to investigate the effects of adding 10% and 30% corn DDGS to the dairy cow diet and to determine the effects of bedding type (wood shavings, straw or peat moss) in dairy slurry on manure fugitive CH_4_ emissions over a 4-month summer storage period. The results of this experiment will guide dairy producers in selecting effective BMPs to attenuate GHG emissions from their operation and ultimately reduce the carbon footprint of dairy products.

## 2. Materials and Methods

### 2.1. Experimental Design

As part of an integrated research project to assess the carbon footprint of milk products in Canada, two experiments were conducted: (1) An animal experiment to evaluate the impact of the level of corn DDGS in Holstein cow’s diets on enteric CH_4_ emissions and on milk yield and composition; (2) A fugitive manure methane emission assessment experiment.

In the animal experiment (used Latin square design), diets containing four different levels of steam-flaked corn DDGS (dry matter-based): 0%—DDGS0, considered as the control diet; 10%—DDGS10; 20%—DDGS20; and 30%—DDGS30 was fed to 16 lactating Holstein cows (645 ± 49 kg) (*i.e.*, 4 diets × 4 cows involved for each diet = 16 cows in each experimental period); four testing periods (duration of each testing period was 4 months) were conducted as per the Latin square design. For the assessment of the effects of diet composition on manure physico-chemical characteristics, urine and feces were collected daily from the 16 cows involved in the 4 diet testing periods.

The collection of feces and urine has been described elsewhere [18]. Briefly, cows were fitted with harnesses and tubes allowing the collection of feces and urine separately. Feces were weighed and mixed daily, and a representative sample (2%) was collected, stored at −20 °C, and subsequently thawed, freeze dried, and ground to pass a 1-mm screen using a Wiley mill for later analysis of DM, VS, total N, NDF, ADF and other parameters. Total urine was collected daily into reinforced plastic containers. The composition of the diets is presented in Table 1 [18].

**Table 1:** Ingredients and composition of the three diets tested—0%, 10% and 30% dried distiller grains with solubles (DDGS0, DDGS10 and DDGS30)—and volume and composition of dairy slurry (kg·day^−1^·cow^−1^) as a function of feeding strategy.

	DDGS0	DDGS10	DDGS30	SEM ^α^	*p*-Value ^&^
**Ingredients, % DM ^β^**					
Alfalfa silage	22.9	22.9	22.9		
Corn silage	33.8	33.8	33.8		
Timothy hay	3.4	3.4	3.4		
Flaked corn	16.7	11.0	0.0		
Soybean meal	13.2	8.8	0.0		
Corn dried distillers grains with	0.0	10.1	30.0		
solubles					
Beet pulp, dehydrated	7.6	7.6	7.5		
Calcium carbonate	0.7	0.7	0.8		
Mineral and vitamin supplement	1.6	1.6	1.5		
**Composition, % DM**					
Organic matter	93.0	92.9	92.5		
Crude protein	16.2	16.4	16.8		
Acid detergent fiber (ADF)	21.8	21.8	23.3		
Starch	19.0	15.8	11.2		
Crude fat	3.99	4.98	7.16		
**Volume and composition of dairy slurry, kg·day^−1^·cow^−1^**					
Slurry	76.1 ^b^	80.2 ^a,b^	84.4 ^a^	2.55	0.0026
Feces	51.9 ^b^	55.2 ^b^	59.8 ^a^	2.33	0.0004
Urine	24.3 ^a^	24.6 ^a^	25.0 ^a^	0.34	0.7151
Dry matter	6.85 ^b^	7.28 ^b^	8.06 ^a^	0.228	<0.0001
Volatile solids	5.98 ^b^	6.39 ^b^	7.05 ^a^	0.203	<0.0001
Nitrogen	0.402 ^b^	0.413 ^a,b^	0.434 ^a^	0.0125	0.0450
Fat	0.433 ^c^	0.557 ^b^	0.737 ^a^	0.0231	<0.0001
Neutral Detergent Fiber (NDF)	3.30 ^b^	3.55 ^b^	4.30 ^a^	0.131	<0.0001
ADF	2.00 ^b^	2.09 ^b^	2.30 ^a^	0.081	0.0013
Hemicelluloses	1.31 ^b^	1.46 ^b^	2.00 ^a^	0.067	<0.0001

^α^ SEM: Standard Error of the Mean; ^&^ *p*-value for diet effect. Within a row, means with a different superscript letter differ significantly (*p* < 0.05).

In the fugitive methane emission assessment experiment, the manure (cow feces and urine) produced from the DDGS0, DDGS10, DDGS30 diets during the first testing period only has been used for assessing fugitive methane emission from manure storage structure. Therefore, for the fugitive methane emission assessment 12 cows were involved (4 cows for each diet × 3 diets (DDGS0, DDGS10, DDGS30)). The manure slurries for each diet were stored in 200-L containers maintained at 4 °C. At the end of the collection period, the slurries from individual diet were homogenized using a portable mechanical mixer and subsamples were taken for analysis to establish the physico-chemical characteristics of manure slurry resulting from each diet.

### 2.2. Incubation Set-Up

#### 2.2.1. Effects of Diet

Storage simulations were performed over a 4-month period in duplicate 54-L Plexiglas storage structures located in a controlled-environment chamber operated at 20 ± 1 °C, in order to simulate the maximum temperature reached in commercial manure storage structures in cold regions like Canada over the summer season (late spring, summer and early fall). Eight storage structures were used to assess the fugitive methane emission from three diets containing three different levels of steam-flaked corn DDGS (dry matter-based): 0%—DDGS0, considered as the control diet; 10%—DDGS10 and 30%—DDGS30. Due to the infrastructure limitation, the diet with 20%—DDGS20 has not been investigated.

Each storage structure initially received 15 kg of psychrophilic residual manure sludge obtained from a storage structure containing dairy cows slurry collected at the Dairy and Swine Research Centre dairy operation. Three sets of duplicate storage structures also received an additional 15 kg of raw slurries from the control, DDGS10 and DDGS30 diets. The average ratio of residual sludge and dairy manure used in this study is representative of commercial scale operation. On commercial farms, when manure storages are emptied, due to the limitation of the manure handling equipment, there is always around 300 mm of residual sludge left at the bottom of the storage structure. When the producer starts to fill the manure storage, the residual sludge to raw manure is very high (around 8) and at the end of the summer the manure ratio of residual sludge to inoculum could be as low as 0.125. For this experiment, we choose a ratio of 1/1. The physico-chemical characteristics of the residual sludge are given in Table 2.

**Table 2:** The physico-chemical characteristics of storage tank residual sludge.

Parameter	Concentration
Volatile fatty acids (VFAs)	48.1 mg·L^−1^
pH	7.48
Dry matter (DM)	34 g·L^−1^
Volatile solids (VS)	24 g·L^−1^
Fixed solids (FS)	10 g·L^−1^
Soluble chemical oxygen demand (SCOD)	6.8 g·L^−1^
Total chemical oxygen demand (TCOD)	37.3 g·L^−1^
N-ammonia (N-NH_3_)	1.8 g·L^−1^
Total Kjeldahl nitrogen (TKN)	2.8 g·L^−1^

In order to determine the relative contribution of the psychrophilic residual manure storage sludge to the CH_4_ emissions, duplicate storage structures received only 15 kg of psychrophilic residual manure sludge.

#### 2.2.2. Effects of Bedding Type

Three types of bedding (wood shavings, straw and peat moss) and a control (without bedding) were tested in duplicate and mixed with 15 kg of slurry from the DDGS0 diet and 15 kg of residual sludge. The mixtures and a control (without bedding) were put in Eight 54-L Plexiglas storage structures located in a controlled-environment chamber operated at 20 ± 1 °C simulating the average storage temperature over a 120-day storage period (late spring, summer and early fall). The quantity of each type of bedding added to the slurry was determined to ensure that the mixture of slurry and bedding reach a dry matter content (DM) of 12%. The corresponding amounts of beddings added were 0.542 kg, 0.509 kg and 0.716 kg, respectively, for wood shavings, straw, and peat moss.

### 2.3. Monitoring Gaseous Emissions

All storage structures were closed hermetically in order to capture and measure daily fugitive biogas emission with wet tip gas meters (a detailed description of the wet tip gas meter is provided on the web site http://wettipgasmeter.com/meters.php). Fugitive biogas samples were collected once a week using a plastic syringe and analyzed to determine the proportion (in %) of CH_4_ in the biogas with Hach Carle 400 AGC gas chromatograph (Model 04131-C, Hach, Loveland, CO, USA) configured for the application 131-C. The application uses a column (1/8 inches) composed of 1.8 m (805 porapak N + 205 Porapak Q), 2.1 m (80% molecular sieve 13X + 20% molecular Sieve 5A), and 1.8 m (80% OV-101 on chromosorb WHP). The column and thermal conductivity detector were operated at 85 °C with a helium gas flow rate of 30 mL·min^−1^ [7]. Calibration was performed weekly with a standard gas (27.3% CO_2_, 1.01% N_2_, 71.69% CH_4_, 0.53% H_2_S).

### 2.4. Chemical Analyses

Feces and urine from the animal experiment were analyzed by the animal team and then summarized for our experiment. The number of samples for the compositional analysis of the dairy slurry was 36 from all testing periods. With regard to the effects of diet on fugitive CH_4_ emissions from manure storage structures, manure samples collected at the start and end of the 120-d laboratory assays were analyzed for pH, DM, VS, FS, SCOD, TCOD (APHA,1992), N-NH3,TKN and VFAs. VFAs (acetic, propionic, butyric, isobutyric, isovaleric) were analyzed by gas chromatography (Perkin Elmer, Norwalk, CT, USA). SCOD and TCOD were determined by the closed reflux colorimetric method (APHA, 1992). SCOD was measured on the supernatant of a centrifuged sample. DM content was determined by drying a 10 g subsample for 24 h at 105 °C. Dried solids were then incinerated for 2 h at 550 °C for volatile content measurement. TKN was analyzed on a subsample digested at 420 °C with selenious acid. TKN and N-NH3 concentrations were determined with a Kjeltec 2400 analyzer (Tecator AB, Hoganas, Sweden). Cell wall fractions (NDF and ADF) of feces were determined by [18] according to the methods of [19] by using a sequential procedure with amylolytic (thermamyl 120 L) treatment.

For the experiment investigating the effects of bedding type on fugitive CH_4_ emissions during storage, wood shavings, straw and peat moss were analyzed with the same analytical methods as indicated in the section above for pH, DM, VS, FS, SCOD, TCOD [20], TKN and VFAs. Samples of fresh bedding types were put in distilled water and kept at 4 °C overnight. The supernatant was then used to analyze the pH, VFAs and SCOD. The other physico—chemical characteristics were measured on 1 mm samples of ground raw material.

### 2.5. Calculations and Statistical Analysis

Data on the volume and composition of the three slurries from the experiment involving 12 dairy cows over the diet testing periods [15] were analyzed with diet as the main effect in a 4 × 4 Latin Square design using the SAS MIXED procedure (SAS release 9.1; SAS Institute Inc., Cary, NC, USA). The composition of the slurries at the beginning and end of the fugitive methane emissions trial were analyzed by ANOVA as a completely randomized design with dietary treatment as the main effect, using the SAS MIXED procedure (SAS release 9.1; SAS Institute Inc., Cary, NC, USA).

CH_4_ production results from the laboratory scale manure storages during the 4-month storage period were also analyzed by ANOVA using the SAS MIXED procedure with diet as the main effect. Multiple means testing was performed using the Tukey test. A significant effect of treatment on least squares means was declared when *p* ≤ 0.05.

Fugitive CH_4_ emissions from storage structures were determined by the following equations:

Daily CH_4_

V CH_4_ (n) = V (n) × CH_4_ (n)

Cumulative CH_4_ over the storage and bioenergy production periods

V CH_4_ = Σ V CH_4_ (n)

Cumulative specific CH_4_ over the storage and bioenergy production periods

Specific V CH_4_ = V CH_4_ cumulated/MVS added

where n is the day the measurement is recorded, V (n) is the volume (L) of biogas, CH_4_ (n) is the percentage of CH_4_ in biogas on that specific day, and MVS is the total mass of volatile solids added (kg). Cumulative V CH_4_ was obtained by summing-up the daily CH_4_ emissions over the 4-month experiment for storage. To express the results in L CH_4_·kg^−1^ VS, the cumulative daily gas emissions expressed in L over the 4-month period for each dietary treatment were first expressed in L CH_4_·kg^−1^ of the mixture (manure slurry + tank residual sludge), then in L CH_4_·kg^−1^ of slurry by subtracting the contribution of the sludge to the daily gas emissions and finally dividing by the percentage of VS excreted per cow.

To express the results in L CH_4_ day^−1^·cow^−1^, the cumulative CH_4_ emissions expressed in L CH_4_·kg^−1^ of slurry were integrated daily, then divided by the number of days of the experiment and multiplied by the amount of slurry excreted daily per cow for each dietary treatment.

## 3. Results and Discussion

### 3.1. Effects of Level of Corn DDGS in the Dairy Cow Diet on Slurry Characteristics

Although the inclusion of corn DDGS had no effect on urine production, dairy cows fed DDGS30 showed a significant increase in the amount of fresh feces and fresh slurry excreted, with increases of 15% (*p* < 0.05) and 11% (*p* < 0.05) per day, respectively, compared to the control diet. The addition of corn DDGS in the dairy cow diet had a significant influence (*p* < 0.05) on some of the physico-chemical characteristics of manure slurry (Table 1). The inclusion of 10% corn DDGS was associated with a significant increase of 29% (*p* < 0.05) in the daily amount of fat excreted in slurry compared to the control diet. The addition of 30% corn DDGS to the diet caused significant increases as follows: 18% in the daily amount of DM (*p* < 0.05); 18% in VS (*p* < 0.05); 70% in fat (*p* < 0.05); 30% in NDF (*p* < 0.05); 15% in ADF (*p* < 0.05); and 53% in hemicellulose (*p* < 0.05) compared to the control diet. There are three possible explanations for the increasing daily amount of slurry excreted: (i) an increase in DM intake tends to decrease nutrient digestion efficiency because feed passes through the digestive system faster; (ii) an increase in water intake moves digestate faster [21]; and (iii) a high degree of lignification of the fiber in the corn DDGS makes it largely inaccessible for degradation by microorganisms present in the digestive tract [22]. It has been reported [23] that manure output usually increases as the concentration of dietary fiber (NDF) increases. This occurs because NDF is generally less digestible than other nutrients. On average, a 1% unit increase in NDF concentration increases manure output by 0.23–0.45 kg·day^−1^. A decrease in fiber digestibility could also explain the increase in the daily amount of DM, VS, NDF, ADF and hemicellulose excreted in connection with the inclusion of 30% corn DDGS.

### 3.2. Effects of the Inclusion of 10 and 30% Corn Dried Distillers in the Dairy Diet on Fugitive CH_4_ Emissions

The initial composition of the slurry (Table 3) showed no difference in the physico-chemical characteristics relative to the control diet, with the exception of the TVFA and the pH of the slurry from the DDGS30 diet; however, statistical analysis was not possible because of the absence of replicates. The statistical analyses of the final composition showed no significant differences in slurry characteristics according to the diet treatment. The TVFA content was zero at the end of the experiment of the three slurries.

**Table 3:** Initial and final composition of slurry and fugitive CH_4_ emissions during the four-month storage experiment as a function of feeding strategies (inclusion of 0, 10%, 30% of corn Dried Distillers Grains with Solubles—DDGS0, DDGS10 and DDGS30).

	DDGS0	DDGS10	DDGS30	SEM ^α^	*p*-Value ^&^
**Initial composition (d.1), g/kg**					
Dry matter (DM, %)	67.2	61.0	63.9		
Volatile solids (VS)	51.8	47.0	49.3		
Fixed solids (FS)	15.4	14.0	14.6		
Total volatile fatty acids (TVFA)	5.67	4.85	4.56		
pH	7.22	7.21	7.31		
Total chemical oxygen demand (TCOD)	80.6	78.1	80.5		
Soluble COD	22.3	25.7	20.9		
Total Kjedahl nitrogen (TKN)	4.21	3.97	4.04		
Ammonia (NH_3_)	2.59	2.46	2.45		
**Final composition (d.120), g/kg**					
DM (%)	52.0 ^a^	49.1 ^a^	50.2 ^a^	1.2	0.3671
VS	36.0 ^a^	34.3 ^a^	34.6 ^a^	0.47	0.4134
TVFA	1.77 ^a^	1.57 ^a^	1.61 ^a^	0.09	0.9016
pH	7.50 ^a^	7.52 ^a^	7.57 ^a^	0.03	0.1153
TCOD	56.7 ^a^	54 ^a^	54.7 ^a^	1.14	0.9872
SCOD	12.3 ^a^	11.2 ^a^	11.2 ^a^	0.52	0.3387
TKN	4.16 ^a^	3.92 ^a^	4.00 ^a^	0.10	0.9810
NH3	3.09 ^a^	2.87 ^a^	2.86 ^a^	0.11	0.4023
**Fugitive CH_4_ emission**					
L·kg^−1^ VS	130.7 ^a^	125.4 ^a^	133.1 ^a^	2.12	0.27
L·day^−1^·cow^−1^	165.7 ^b^	172.3 ^b^	190.1 ^a^	25.35	0.013

^α^ SEM: Standard Error of the Mean; ^&^ *p*-Value for Diet effect. Means on the same line with different superscript letters differ significantly (*p* < 0.05).

**Figure 1:**
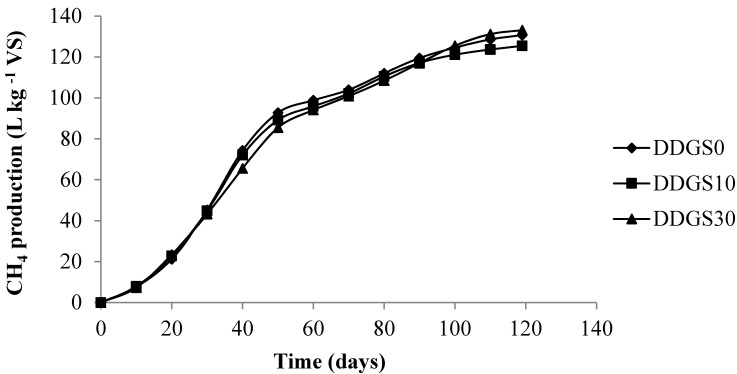
Effects of feeding strategy (adding 0%, 10%, 30% of corn dried distillers’ grains with solubles (DDGS)) on manure CH_4_ emissions (L CH_4_·kg^−1^ volatile solids (VS)) over a 4-month storage experiment.

When compared with the 0% corn DDGS diet, the addition of corn DDGS did not have a significant effect on cumulative fugitive CH_4_ emissions per kg of VS (Table 2 and Figure 1). The profiles of the cumulative fugitive CH_4_ production were similar over the four month-storage experiment for all the treatments. However, the total daily fugitive CH_4_ emissions (L CH_4_·day^−1^·cow^−1^) showed a significant increase of 15% (*p* < 0.05) in the slurry from the DDGS30 diet compared to the control diet. This can be explained by the fact that the addition of DDGS30 to the diet caused a significant increase (*p* < 0.05) in the daily amount of feces and slurry excreted. The increase in the masses of feces, VS, fat and hemicellulose excreted were 15%, 17.9%, 70%, and 53% respectively. In this study, fugitive CH_4_ emissions depend mainly on the amount of VS excreted.

### 3.3. Effects of Different Bedding Types in Dairy Cow Liquid Manure Slurry on Fugitive CH_4_ Emissions during a 4 Month Storage Experiment

The composition of the bedding types is provided in Table 4.

**Table 4:** Composition of the bedding types used to assess fugitive methane emissions from slurry with bedding at 20 °C.

	Wood Shavings	Straw	Peat Moss
Composition, % DM			
Dry matter (DM, %)	93	94	57
Volatile solids (VS)	28	28	32
Fixed solids (FS)	72	72	68
Total volatile fatty acids (TVFA)	0.02	0.44	0.30
pH	5.26	7.55	5.11
Total chemical oxygen demand (TCOD)	126	117	186
Soluble COD	1.47	3.45	0.88
Total Kjedahl nitrogen (TKN)	0.22	0.48	1.16
Ammonia (NH_3_)	0.00	0.04	0.08

The effect of bedding type on the cumulative emissions of fugitive CH_4_ during the 4-month storage experiment is shown in Table 5. The results showed a significant effect of bedding type on the fugitive CH_4_ emissions.

Emissions of CH_4_ per kg of VS for the slurry with sludge showed significant reductions (*p* < 0.05) of 30%, 23% and 13% with the addition of wood shavings, straw and peat moss, respectively (Table 5), compared to the control without bedding. When the fugitive CH_4_ emissions were expressed in relation to the total amount of VS excreted per cow daily and VS added to the bedding, the addition of wood shavings and straw did not significantly affect fugitive CH_4_ emissions, unlike the addition of peat moss, which caused a significant increase of 27% (*p* < 0.05) in CH_4_ emissions compared to the control without bedding. Therefore, the use of peat moss as bedding is not recommended for open storage structures because it enhances fugitive CH_4_ emissions. We also observed the formation of a crust on slurries with the addition of straw and wood shavings. Although this experiment was not designed to investigate the effect of crust formation on fugitive CH_4_ emissions, such a crust could create a barrier that limits access of the microbial biomass to wood shavings and straw.

**Table 5:** Effects of including three bedding types (wood shavings, straw and peat moss) in dairy slurry from the controlled experiment (0% DDGS) on manure CH_4_ emissions during 4 months of storage.

Fugitive CH_4_ Emission	0% Corn DDGS ^α^	0% Corn DDGS ^α^ +	0% Corn DDGS ^α^ +	0% Corn DDGS ^α^ +	SEM ^γ^	*p-*Value
		Wood shavings ^β^	Straw ^β^	Peat moss ^β^		
L·kg^−1^ VS	130.7 ^a^	91.38 ^c^	100.9 ^b c^	113.6 ^b^	14.7	0.002
L·day^−1^·cow^−1^	165.7 ^b^	163.2 ^b^	167.1 ^b^	210.3 ^a^	19.5	0.004

^α^ DDGS = dried distillers grains with solubles; ^β^ Quantity of bedding type incorporated into 15 kg of liquid manure was 0.542 g for wood shavings, 0.509 g for straw and 0.716 g for peat moss, in order to reach a DM content of around 12%. ^γ^ SEM: Standard Error of the Mean, Diet: *p*-value for diet effect. Within a row, means with a different superscript letter differ significantly (*p* < 0.05).

## 4. Conclusions

The addition of corn dried distillers grains with solubles (DDGS) to the dairy cow diet changed the manure slurry characteristics and increased the level of fugitive methane emission from manure storage structures. The inclusion of DDGS30 in the diet increased the daily amounts of excreted DM, volatile solids (VS), fat, NDF, ADF and hemicellulose by 18%, 18%, 70%, 30%, 15% and 53%, respectively, compared to the control diet. It also significantly increased the daily fugitive CH_4_ emissions from manure slurry by 15% (*p* < 0.05). The contribution of bedding type (wood shavings, straw or peat moss) to manure slurry fugitive CH_4_ emissions is different. The addition of wood shavings and straw did not have a significant effect on daily fugitive CH_4_ emissions in relation to the control diet, whereas the addition of peat moss was associated with a significant increase (27%) in fugitive CH_4_ emissions (*p* < 0.05). The choice of bedding type should be taken into account in order to reduce fugitive CH_4_ emissions. These data will be used to carry out a detailed and comprehensive life cycle analysis of Canadian dairy products.
